# Assessing the Multiple Dimensions of Poverty. Data Mining Approaches to the 2004–14 Health and Demographic Surveillance System in Cuatro Santos, Nicaragua

**DOI:** 10.3389/fpubh.2019.00409

**Published:** 2020-01-29

**Authors:** Carina Källestål, Elmer Zelaya Blandón, Rodolfo Peña, Wilton Peréz, Mariela Contreras, Lars-Åke Persson, Oleg Sysoev, Katarina Ekholm Selling

**Affiliations:** ^1^Department of Women's and Children's Health, Uppsala University, Uppsala, Sweden; ^2^Asociación para el Desarrollo Económico y Sostenible de El Espino (APRODESE), Chinandega, Nicaragua; ^3^Nicaraguan Autonomous National University, León (UNAN-León), León, Nicaragua; ^4^Pan American Health Organization, Tegucigalpa, Honduras; ^5^Department of Disease Control, London School of Hygiene & Tropical Medicine, London, United Kingdom; ^6^Department of Computer and Information Science, Linköping University, Uppsala, Sweden

**Keywords:** multidimensional poverty, capability approach, health and demographic surveillance, data mining, K-means clustering, poverty alleviation

## Abstract

We identified clusters of multiple dimensions of poverty according to the capability approach theory by applying data mining approaches to the Cuatro Santos Health and Demographic Surveillance database, Nicaragua. Four municipalities in northern Nicaragua constitute the Cuatro Santos area, with 25,893 inhabitants in 5,966 households (2014). A local process analyzing poverty-related problems, prioritizing suggested actions, was initiated in 1997 and generated a community action plan 2002–2015. Interventions were school breakfasts, environmental protection, water and sanitation, preventive healthcare, home gardening, microcredit, technical training, university education stipends, and use of the Internet. In 2004, a survey of basic health and demographic information was performed in the whole population, followed by surveillance updates in 2007, 2009, and 2014 linking households and individuals. Information included the house material (floor, walls) and services (water, sanitation, electricity) as well as demographic data (birth, deaths, migration). Data on participation in interventions, food security, household assets, and women's self-rated health were collected in 2014. A K-means algorithm was used to cluster the household data (56 variables) in six clusters. The poverty ranking of household clusters using the unsatisfied basic needs index variables changed when including variables describing basic capabilities. The households in the fairly rich cluster with assets such as motorbikes and computers were described as modern. Those in the fairly poor cluster, having different degrees of food insecurity, were labeled vulnerable. Poor and poorest clusters of households were traditional, e.g., in using horses for transport. Results displayed a society transforming from traditional to modern, where the forerunners were not the richest but educated, had more working members in household, had fewer children, and were food secure. Those lagging were the poor, traditional, and food insecure. The approach may be useful for an improved understanding of poverty and to direct local policy and interventions.

## Introduction

The first of the Sustainable Development Goals aims at ending poverty in all its forms, everywhere. This is further specified as reducing by 2030 at least by half the proportion of men, women, and children of all ages that currently live in poverty *in all its dimensions according to national de*finitions (our underscore) (1). This all-inclusive target addresses all dimensions of poverty, the most important determinant for health and well-being (2).

The poverty measures used by the World Bank and many international agencies are usually monetary measures on the national level, such as the poverty line at 1.90 purchasing power parity dollar and the gross domestic product per capita. These monetary measures of poverty are possible to compare over time and across nations. In Latin America, the unsatisfied basic need (UBN) index has been widely used to compare poverty at the household level between different geographical areas (3, 4). UBN is a composite index that includes housing conditions, access to water and sanitation, school enrolment, education of the head of the household, and the ratio of dependent household members to working-age members. In the Demographic Health Surveys, household asset scores have been widely used as a measurement of household socioeconomic position and poverty (5). Asset scores have been used to stratify other outcomes along a wealth axis, such as the identification and explanation of social inequalities in health (6). Asset scores cannot be used to follow or compare development over time since each index is only valid for the survey for which it was created.

The Commission on Global Poverty, assigned by the World Bank (7), recommended the inclusion of complementary indicators when tracking poverty change over time and across settings. Further, the Commission suggested the capability approach to poverty formulated by Amartya Sen and others as a framework to aid the development of indicators (8, 9).

**Figure 1:**
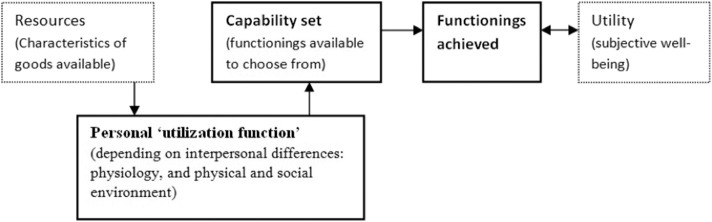
Outline of the core relationships in the capability approach. Source: The Internet Encyclopedia of Philosophy (https://www.iep.utm.edu/sen-cap/).

The capability approach focuses on individuals and prioritizes the freedom of choice a person has over alternative lives that she or he could live (9). This approach deals with the potential to choose when answering the question, “What is this person able to do and be?” (10). Capabilities allow freedom of action and decisions, i.e., opportunities of life choices and thus well-being. In this approach, the fundamental and intertwined concepts are capabilities and functions. Functions are states as being well-nourished and having shelter, while capabilities are a set of functions that the person has access to, or the realization of capabilities (11). Figure 1 illustrates the concepts.

In practice, it is often easier to evaluate achieved functions, representing the accomplished or chosen capabilities. People show adaptive preferences to their environment by adjusting their expectations to the surrounding social, cultural, political, and economic restrictions (personal utilization function in Figure 1). Therefore, frequently capabilities cannot be converted to functions, thus indicating the need for equality in capabilities and functioning (12).

Amartya Sen and others have discussed whether basic capabilities should be captured in indices or decided upon by the poor themselves (8). The interest in identifying a list lies in the possibility it gives to evaluate well-being or the lack thereof as expressions of poverty. In most cases when such basic capabilities are listed, the basic capabilities included are adequate health, sufficient food and nutrition, adequate education to ensure basic knowledge, the capability of independent thought and expression, political participation, and freedom of race, religion, and gender discrimination (12). Hence, several indices that capture multiple basic capabilities have been developed in order to map the situation and incite policy action, such as the Multidimensional Poverty Index (13).

Governments have the responsibility to implement policies for poverty reduction to reach the first Sustainable Development Goal (14). Local level bottom-up interventions might, however, result in sustainable poverty reduction that inspires decision-makers at the national level. We have documented such a case in northern Nicaragua: the Cuatro Santos experiences of local poverty reduction (15). That case study showed that factors such as local ownership, locally guided multidimensional interventions, and close monitoring and evaluation of the development efforts yielded a substantial poverty reduction of household poverty from 79% to 47% over 10 years (2004–2014) (15).

In the Cuatro Santos area, a Health and Demographic Surveillance System (HDSS) was established in 2004 with the latest update in 2014. Participation in microcredit programs, the involvement of young individuals in technical training, and home gardening were associated with the transition of households out of poverty (16). The UBN scoring of households was used to identify geographic areas with higher levels of poverty to target interventions (15). However, poverty indices, such as the UBN or asset scores, have limitations for a context-specific description of poverty sufficiently detailed for directing interventions aiming to increase well-being and equity. The UBN index is a score including seven variables describing the household's services and conditions, economic capacity, and school enrolment, which do not capture all dimensions of poverty, especially since house conditions and service might remain for a long time irrespective of the household's change of poverty status.

A data mining method, a variant of the K-means clustering algorithm (17), is an alternative method. This method has the power to identify patterns, which describe the multiple dimensions of poverty in a local context given that a sufficient number of variables measuring basic capabilities are at hand, e.g., when HDSS data exist. This method allows for many more variables to be included; thus, it catches more dimensions of a household's status. When more variables are at hand, more dimensions of poverty can be described as suggested in the capability theory. Such a description infers the possibility of identifying recipients of interventions or general local policy actions for the reduction of poverty.

Thus, this paper aims to describe the multiple dimensions of poverty according to the capability approach theory by applying the K-means clustering method to the Cuatro Santos Health and Demographic Surveillance databases, Nicaragua.

## Methods

### Study Setting, Population, and Design

**Figure 2:**
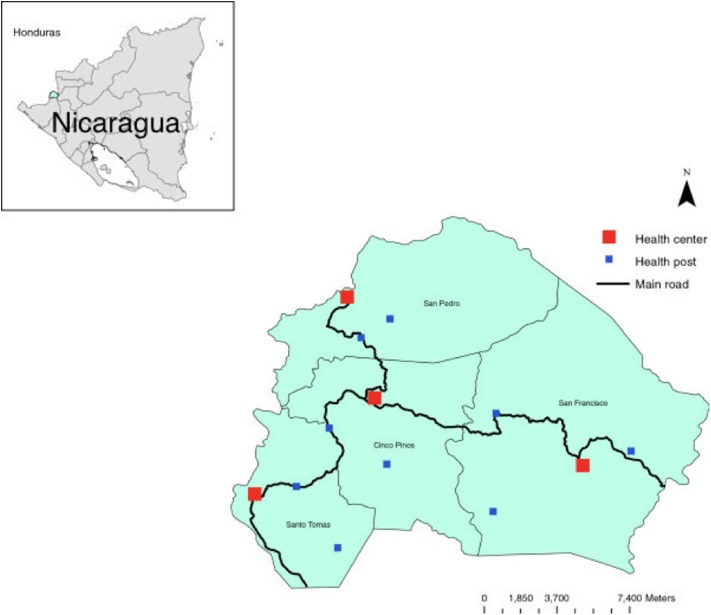
The Cuatro Santos area showing the four municipalities and health facilities. The area is marked in the inserted Nicaragua map.

The Cuatro Santos area, situated in the northern part of Chinandega, Nicaragua, consists of four municipalities of similar population size. In 2014, 25,893 inhabitants lived in 5,966 households in an area located 250 km northwest of the capital of Nicaragua, Managua, in a mountainous terrain bordering Honduras. The climate is predominantly dry, and traditional sources of income have been the cultivation of grains and raising livestock, now with an increasing number of small-scale enterprises. This area was strongly affected by the Contras war in the 1980s and the hurricane Mitch in October 1998. Since that time, a significant proportion of the population has out-migrated due to economic reasons, including fixed or seasonal work or search for employment (18). A map of the area is shown in Figure 2.

### Community Interventions in Cuatro Santos

Starting in 1997, representatives of the four municipalities, the local non-governmental organizations, local government leaders, and representatives of national institutions initiated a process labeled “decoding reality,” which was inspired by Paulo Freire (19). This process included an analysis of the local poverty-related problems, prioritization among suggested actions, and an action plan that was approved as the Cuatro Santos Area Development Strategy 2002–2015. This strategy aimed at developing the area by use of local resources, informed by data from the surveillance system, and to attract international cooperation. The concepts of local ownership and participation were central, and the efforts included consensus decision-making and reconciliation in case of conflicts. Priority interventions were school breakfasts, environmental protection, water and sanitation, preventive healthcare, home gardening, microcredit, technical training, stipends for university education, and telecommunications including access to and training in the use of the Internet. Data collection through a HDSS was central for monitoring of trends over time and research evaluation of various aspects (15, 16).

### Cuatro Santos Health and Demographic Surveillance System

In 2004, a census and cross-sectional data collection of basic health and demographic information was performed in the whole population. Follow-up surveys were performed in 2007, 2009, and 2014. Unique identifiers of households and individuals linked the data. Demographic changes in households, such as birth, death, and migration, were registered. Household data included information on the house (floor, walls) and services (water, sanitation, electricity). All women aged 15–49 years living in households provided retrospective reproductive histories (16). In the 2009 and 2014 updates, questions were included on participation in the following interventions: access to water and latrines, microcredit, home gardening, technical education, school breakfast programs, and telecommunications. In the 2014 update, data on food security, household assets, and women's self-rated health were collected. For the present study, data from the 2014 update were used, including information on earlier interventions mainly deployed during 2005–2009 (16).

Fieldwork was conducted by local female fieldworkers who were carefully supervised, printed forms were checked before computerization, and the forms were returned to the field if the information was missing or suspected to be incorrect. Further data quality controls were completed after computerization, including logical controls. Data were carefully cleaned and stored in a relational database (Microsoft Access 2007®).

### Variables (see Table 1)

Persons residing in a household at the time of the field survey defined the household. Migration was defined as a household member aged 18–65 who migrated in or out of the household since the previous update (5 years). The UBN index (5) was composed of four components: (1) housing conditions (unsatisfied: walls of wood, cardboard, plastic, or earthen floor); (2) access to water and latrine (unsatisfied: water from river, well, or bought in barrels and no latrine or toilet); (3) school enrolment of children (unsatisfied: any children 7–14 years of age not attending school); and (4) education of the head of the family and the ratio of dependent (<15 and >65 years) household members to working-age members (15–65 years) (unsatisfied: head of the family illiterate or dropped out of primary school and ratio of dependent household members to working-age members > 2.0). Each component rendered a score of 0 if satisfied and 1 if unsatisfied. Thus, the total sum varied from 0 to 4. Households with 0 or 1 UBN were considered non-poor, while poor households had 2–4 UBN. Characteristics of houses and households were also included in the cluster analyses, such as the material of walls, floor, access to electricity, type of stove, access to water, and type of toilet. The interventions implemented in the area were represented by household-related information on such participation. The presence of a water meter indicated that the household had got water installed as part of the last decade's interventions. Also, information was included on previous and current participation in home gardening, if anyone in the household had received microcredits or had participated in technical training.

**Table 1:** List of variables included in the analyses of Cuatro Santos database, Nicaragua 2014, including descriptive statistics.

**Categorical variables**	**Labels**	***n***	**%**
Poverty	0 Not poor = UBN 0–1	2,828	53.8
	1 Poor = UBN 2–4	2,425	46.2
Unsatisfied basic needs (UBN)	0 No basic need unsatisfied	1,161	22.1
	1 Wall is made of wood, cartons, plastic, AND mud floor	1,667	31.7
	2 Access to water is through rivers, wells, or bought in barrels AND no latrine	2,167	41.3
	3 Children ages 7–14 years are not attending school	251	4.8
	4 The head is illiterate or not completed primary school AND dependency ratio>2	7	0.1
House wall type	1 Ceramic brick	1,465	27.9
	2 Adobe/wattle wall	3,707	70.6
	3 Wood	31	0.6
	4 Palm	3	<0.1
	5 Cardboard, plastic, metal	42	0.8
	6 Without walls	5	<0.1
Water availability	1 Indoor pipe	1,807	34.4
	2 Commune post	117	2.2
	3 Own well	1,117	21.3
	4 Communal well	1,538	29.3
	5 River/creek	410	7.8
	6 Purchased water	6	0.1
	7 Other sources	258	4.9
Toilet type	1 Toilet	133	2.5
	2 Latrine	4,123	78.5
	3 No toilet/latrine	997	19.0
Floor in house	1 Ceramic brick	418	8.0
	2 Brick/cement	272	5.2
	3 Mud brick	42	0.8
	4 Tiling	1,567	29.8
	5 Mud floor	2,954	56.2
Electricity in house	1 Yes	4,683	89.1
	2 No	570	10.8
Stove in house	1 Gas	469	8.9
	2 Wood/improved	75	1.4
	3 Wood/normal	4,664	88.8
	4 Does not have	45	0.9
Water meter in use	1 Yes	1,130	21.5
	2 No	4,123	78.5
Microcredits in HH^*^	1 Yes	671	12.8
	2 No	4,582	87.2
Technical training in HH^*^	1 Yes	514	9.8
	2 No	4,739	90.2
Home garden in HH^*^	1 Yes	321	6.1
	2 No	4,932	93.9
Home garden in use	1 Yes	197	3.8
	2 No	5,056	96.2
Anxiety in HH^*^ for lack of food	0 Never	705	13.4
	1 Rarely (1–2 times)	2,106	40.1
	2 Sometimes (3–10 times)	1,303	24.8
	3 Often (>10 times)	1,139	21.7
Inability in HH^*^ to eat preferred food	0 Never	692	13.2
	1 Rarely (1–2 times)	2,216	42.2
	2 Sometimes (3–10 times)	1,803	34.3
	3 Often (>10 times)	542	10.3
Limited variation of food in HH^*^ due to lack of food	0 Never	989	18.8
	1 Rarely (1–2 times)	2,421	46.1
	2 Sometimes (3–10 times)	1,440	27.4
	3 Often (>10 times)	403	7.7
Few kinds of food consumed in HH^*^ due to lack of food	0 Never	896	17.1
	1 Rarely (1–2 times)	2,584	49.2
	2 Sometimes (3–10 times)	1,427	27.2
	3 Often (>10 times)	346	6.6
Reduction of portion sizes of meals in HH^*^ due to lack of food	0 Never	1,307	24.9
	1 Rarely (1–2 times)	2,524	48.0
	2 Sometimes (3–10 times)	1,166	22.2
	3 Often (>10 times)	256	4.9
Fewer meals consumed in HH^*^ due to lack of food	0 Never	2,016	38.4
	1 Rarely (1–2 times)	2,167	41.3
	2 Sometimes (3–10 times)	892	17.0
	3 Often (>10 times)	178	3.4
No food to eat in HH^*^ due to lack of resources	0 Never	3,734	71.1
	1 Rarely (1–2 times)	1,132	21.5
	2 Sometimes (3–10 times)	335	6.4
	3 Often (>10 times)	52	1.0
HH^*^ going to sleep hungry due to lack of food	0 Never	4,478	85.2
	1 Rarely (1–2 times)	564	10.7
	2 Sometimes (3–10 times)	189	3.6
	3 Often (>10 times)	22	0.4
HH^*^ having days of hunger due to insufficient amount of food	0 Never	4,744	90.3
	1 Rarely (1–2 times)	367	7.0
	2 Sometimes (3–10 times)	124	2.4
	3 Often (>10 times)	18	0.3
TV antenna in HH^*^	1 Parabolic antenna	604	11.5
	2 Normal antenna	2,069	39.4
	3 Handmade antenna	429	8.2
	4 No antenna	2,151	40.9
Car in HH^*^	1 Yes	137	2.6
	2 No	5,116	97.4
Motorbike in HH^*^	1 Yes	443	8.4
	2 No	4,810	91.6
Bike in HH^*^	1 Yes	872	16.6
	2 No	4,381	83.4
Horse in HH^*^	1 Yes	1,347	25.6
	2 No	3,906	74.4
Refrigerator in HH^*^	1 Yes	1,567	29.8
	2 No	3,686	70.2
Sewing machine in HH^*^	1 Yes	337	6.4
	2 No	4,916	93.6
Computer in HH^*^	1 Yes	183	3.5
	2 No	5,070	96.5
Tortilla oven in HH^*^	1 Yes	916	17.4
	2 No	4,337	82.6
Stove with chimney in HH^*^	1 Yes	103	2.0
	2 No	5,150	98.0
Deaths in HH^*^	0 No deaths in HH^*^	4,934	93.9
	1 Deaths in HH^*^	319	6.1
Births in HH^*^	0 No births in HH^*^	3,907	74.4
	1 Births in HH^*^	1,346	25.6
Immigration in HH^*^	0 No immigration in HH^*^	3,206	61.0
	1 Immigration in HH^*^	2,047	39.0
Emigration in HH^*^	0 No emigration in HH^*^	2,289	43.6
	1 Emigration in HH^*^	2,964	56.4
Sex of HH head	1 Female head of HH^*^	1,382	26.3
	2 Male head of HH^*^	3,871	73.7
Illiterate living in HH^*^	0 No illiterate in HH^*^	3,812	72.6
	1 Illiterate in HH^*^	1,441	27.4
Highest education in HH^*^	0 No education	208	4.0
	2 Primary school	1,679	32.0
	3 Secondary school	2,312	44.0
	4 Technical education	379	7.2
	5 University education	675	12.8
HH^*^ member immigrated from foreign country	0 No immigration from another country in household	4,928	93.8
	1 Immigration from another country in HH^*^	325	6.2
HH^*^ member emigrated to foreign country	0 No emigration to another country in HH^*^	4,560	86.8
	1 Emigration to another country in HH^*^	693	13.2
Children (<15 years) in HH^*^ working	0 No	5,172	98.4
	1 Yes	81	1.5
Home birth in HH^*^	0 No home birth in HH^*^	5,143	97.9
	1 Home birth in HH^*^	110	2.1
Hospital birth in HH^*^	0 No hospital birth in HH^*^	4,153	79.1
	1 Hospital birth in HH^*^	1,100	20.9
Child health center birth in HH^*^	0 No CHC birth in HH^*^	4,892	93.1
	1 CHC birth in HH^*^	361	6.9
Under 5 death in HH^*^	0 No	5,195	98.9
	1 Yes	58	1.1
Women's self-rated health in HH^*^	0 No women with bad health in HH^*^	2,963	56.4
	1 Women with bad health in HH^*^	2,290	43.6
	**Mean (Median)**	**Min**	**Max**
**CONTINUOUS VARIABLES**			
No. of children in HH^*^	1.7 (2.0)	0	12
No. of adults in HH^*^	4.7 (4.0)	0	19
No. in HH^*^ not working	2.6 (2.0)	0	13
No. in HH^*^ working	1.4 (1.0)	0	9
No. of working adults (≥15 years) in HH^*^	1.4 (1.0)	0	9
No. of not working adults (≥15 years) in HH^*^	1.7 (1.0)	0	8
No. of individuals in HH^*^	6.5 (6.0)	1	25
Ratio of adults working to not working in HH^*^	1.6 (1.0)	0	9
Ratio of working adults (≥15 years) to no. of individuals in HH^*^	0.2 (0.2)	0	1

* *HH, household*.

A nine-item Household Food Insecurity Access Scale (HFIAS), version 3, was used (20). The respondents were either the head of the household or the person responsible for the household expenditure and food preparation during the last four previous weeks. This scale covers experiences regarding (1) anxiety in the household due to lack of food; (2) inability to eat preferred food because of lack of resources; (3) limited variety of food due to lack of resources; (4) consumption of a few kinds of food because of lack of resources; (5) reduction of portion sizes of meals due to lack of food; (6) consumption of fewer meals per day because of lack of food; (7) no food to eat in the household because of lack of resources; (8) going to sleep at night hungry due to lack of food; and (9) days of hunger because of insufficient amounts of food to eat. For each affirmative answer, the person provided additional information on the frequency in a four-point scale (never, rarely, sometimes, often).

Household assets were TV antenna, car, motorbike, bike, horse, refrigerator, sewing machine, computer, tortilla oven, and a chimney for the wood-burning stove.

The individual variables collected in 2014 were derived and aggregated at the household level, and after that merged with the variables at the household level. We constructed variables on births and deaths in the household during the recent update period, also including information on under-5 death, the number of adults and children living in the household, the number of adults and children working, the number of adults not working, and the ratio between adults working and not working, as well as the ratio between adults working and the number of individuals in the household. Further, data were included on in- and out-migration, including from foreign countries, the gender of household head, any illiteracy, and the highest education level in the household (none, primary, secondary, technical, university education). Information was also included if a home, health center, or hospital birth had happened since the last update (5 years).

Women's self-rated health was assessed for all resident women of reproductive age (15–49 years) at the time of the interview by a five-point Likert scale based on the following question: “In general, how would you assess your health today?” The interviewer provided the following options: very good, good, medium, bad, or very bad. This information was classified as good (very good, good, medium) or bad (bad, very bad) health. No household had a mix of good and bad self-assessed health when aggregating this information to the household level. The entire dataset included 56 variables.

### Analytical Methods

All analyses were performed on the household level. The variables included are displayed in Table 1. A variant of the K-means clustering algorithm (17) called SimpleKMeans in Weka (21) was used to perform a clustering of the data. The reason for choosing the K-means algorithm was that K-means is “the most popular and the simplest partitional algorithm” (22). The K-means algorithm computes K points called centroids and then assigns the data points to their respective closest centroid. This leads to forming K groups (clusters) of observations in the data where observations within each cluster have similar properties. SimpleKMeans algorithm differs from the original k-means algorithm in the strategy of choosing initial centroids. While in the original k-means algorithm the initial centroids are selected randomly, SimpleKMeans algorithm generates initial centroids sequentially as follows. When c centroids are generated, centroid c+1 is sampled as a data point from the data set with probability proportional to the distance to the closest existing centroid. This strategy places initial centroids relatively far from each other. Compared to the original k-means algorithm that has no theoretical guarantees on the quality of clustering, this alternative centroid selection strategy has been shown to lead to a guaranteed theoretical and also improved practical clustering quality in different settings (23). To evaluate the quality of the clustering, data were split into training and test sets. Cluster centroids were computed from the training data and tested on the test data by using the closest centroid principle. Properties of the training and test clusters were compared, and the robustness was evaluated.

Categorical variables were transformed into dummy variables and included in the K-means cluster analysis, and after being scaled, the numerical variables were also included in the analysis. Repeated analyses where performed forcing data into 2–10 clusters. Default values were taken for all other settings of the algorithm. A so-called scree plot was created displaying cluster sums of squared errors (y-axis) and the number of clusters (x-axis) (Supplemental Figure 1). An appropriate number of clusters in the plot can be found by identifying the level of the x variable where the saturating starts. Six clusters were selected after inspection of this scree plot and checking cluster sizes. The Euclidian distance was applied, and the data were randomly split into training (66%) and test (34%) sets. The meaning of the clusters was interpreted by evaluating the cluster centroids (percentages for dummy variables of categorical variables and averages for numerical variables) in each cluster in relation to each other and the full data.

Groups of variables with similar characteristics were analyzed in a stepwise manner to generate an assessment of poverty. These groups of variables were included in the following order: (a) poverty assessed by the variables poverty and UBN and variables in UBN except head of household's education, children's school enrolment, and the ratio of dependents to working household members; (b) assets; (c) food insecurity; (d) interventions; and (e) derived individual variables (see Table 1 for included variables and Supplemental Table 1 for full cluster analysis output where the variable groups are color marked). The emerging patterns were evaluated, and the clusters were labeled in words as reported in the results. Table 2 shows the essential variables extracted from Supplemental Table 1, yielding the labeling words. A first ranking of clusters from the poorest to the richest was made using the variable group a) poverty and UBN (Table 3), which was changed into a new ranking taking all groups of variables into consideration (Table 4).

**Table 2:**
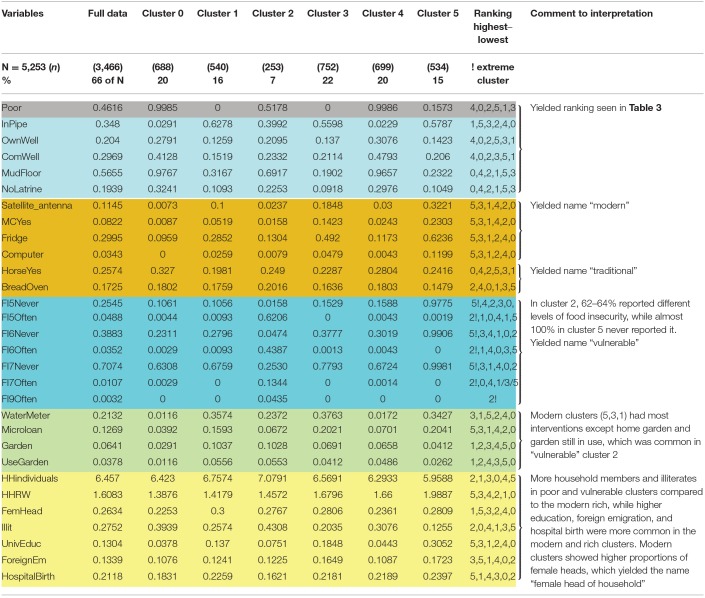
Meaningful variables used in the analysis of clusters illustrating naming of clusters.

*Extracted from Supplemental Table 1, categories color marked as follows: Gray, Poverty assessed by the variable poverty; Light blue, variables in UBN, except head of household's education, children's school enrolment, and dependency ratiol; Dark yellow, assets; Turquoise, food insecurity; Green, interventions; Light Yellow, derived individual variables*.

**Table 3:** Results from cluster analysis of first ranking using Unsatisfied Basic Needs (UBN) variables from the Health and Demographic Surveillance System, Cuatro Santos, Nicaragua.

**Cluster** **(% of HH^b^)**	**Poverty^a^**
4 (20%)	Poorest
0 (20%)	Poor
2 (7%)	Fairly poor
5 (15%)	Fairly rich
1(16%)	Rich
3 (22%)	Richest

a *Rich and poor refer to our UBN categories and household characteristics included in the UBN*.

b *HH, households*.

**Table 4:** Results from cluster analysis second ranking including all variables from the Health and Demographic Surveillance System, Cuatro Santos, Nicaragua.

**Cluster** **(% of HH^b^)**	**Multidimensional poverty^a^**
2 (7%)	Fairly poor, most vulnerable, fairly traditional
0 (20%)	Poor, traditional
4 (20%)	Poorest, traditional
1 (16%)	Rich, fairly modern, female head of household
3 (22%)	Richest, fairly modern, female head of household
5 (15%)	Fairly rich, most modern, female head of household

a *Rich and poor refer to our Unsatisfied Basic Needs (UBN) categories and household characteristics included in the UBN, while modern and traditional refer to assets, interventions, number of adults and children in the household, education, emigration, and hospital births. Vulnerable refers to food security and female head of the household to the proportion of female-headed households*.

b *HH, households*.

### Ethical Considerations

The information was collected as part of the Health and Demographic Surveillance update survey in 2014. The Ethical Review Board of Biomedical Research at the National Autonomous University of León approved the HDSS data collection (FWA00004523/IRB0000334 ACTA No. 81). Informed verbal consent was obtained from the participants. They were free to end their participation at any time. Data were stored in a safe electronic platform with an alphanumeric identification number instead of names of participants to protect confidentiality.

## Results

Of the 5,966 households included in the 2014 update of the HDSS, 5,253 (88%) were included in the analyses after eliminating households with missing values on any variable. The primary reasons to omissions were houses included in the database as households while, in fact, not being living quarters, e.g., schools, health centers, or abandoned houses. Included data measured experiences since the last update (5 years), earlier participation in interventions, and recent food insecurity and self-rated health. The basic characteristics of households are shown in Table 1.

### Cluster Analyses

The patterns emerging from the essential variables extracted from Supplemental Table 1 and the labeling of clusters are illustrated in Table 2.

The first ranking of clusters was based on the earlier used UBN index for enabling comparisons to further analysis, including more variables. Poverty assessed by the first group of variables, i.e., the dichotomized variable poverty and the five UBN categories (0–4), and the variables characterizing the household physical conditions and the water and sanitation conditions (Supplemental Table 1 and Table 2) yielded a ranking by *poverty status* as shown in Table 3 with variables separating the clusters the most (in the following text, these variables are called essential variables), being “poor,” “water source,” “mud floor,” and “no latrine.”

When including the variables in the assets group of variables, cluster 5 (Table 2) is shown to be the most *modern* cluster having assets that were modern equipment like “satellite dish antenna,” “computer,” “refrigerator,” and “motorbike.” Clusters 1 and 3 also had these assets but to a lesser extent. Clusters 0, 2, and 4 were more *traditional* with assets such as “horses” and “tortilla bread ovens” in higher proportions, illustrating that transportation and earnings of living by selling tortillas were carried out as in earlier times. These assets yielded the names *traditional* and *modern*.

In the following step, variables in the food insecurity group of variables were analyzed. The distribution of food insecurity variables showed that cluster 2 (7% of households) was far more food insecure than all other clusters, including all aspects of food security and that cluster 5 was food secure. These characteristics added the descriptive word *vulnerable*.

When proceeding to variables in the interventions group of variables, the results showed that the most modern, richest, and least vulnerable cluster had participated most in the interventions. One exception was home gardening and still using a garden, which was more common among the traditional and vulnerable clusters, especially the food insecure cluster 2. The latter intervention had, however, reached few households. The essential variables were “water meter,” “microcredit”, “technical training,” and “home gardening.”

When including all variables, the re-ranking displayed clusters of *multidimensional poverty*, and the derived individual variables made this new ranking more distinct (Table 4).

More household members and children were found in the poor and vulnerable clusters compared to the modern rich, while higher education was more common in the modern and rich clusters. Overall, female- and male-headed household proportions were ¼ and ¾, respectively, and the more modern clusters showed higher proportions of female heads, which rendered the descriptive word *female head of household* in the naming of clusters. The following were the most essential of the derived individual variables: “number of household individuals,” “ratio of adults working to those not working,” “female/male household head,” “illiterate individuals in household,” “university education in household,” “foreign emigration in household,” and “hospital birth,” which all strengthened the multidimensional poverty group ranking and modern or traditional labeling.

## Discussion

This study is unique as it assesses the multiple dimensions of poverty based on data mining technique using data at the household level with a large number of variables. The household level was chosen as the level of measurement as it is in demographic surveillance. Variables assessing household conditions, food insecurity, access to interventions, and demographic events such as mortality were used. We found six clusters of households with differences between them and similarities within them, based on their shared variables.

We first ranked clusters of the households as being more or less poor and rich, using the UBN index variables in the cluster analysis. This ranking was changed when including more variables describing basic capabilities. Most importantly, the fairly rich cluster (cluster 5) showed to be the most modern, with modern assets such as motorbikes and computers. The fairly poor cluster (cluster 2) showed to be the most vulnerable, having varying degrees of food insecurity, something that the most modern cluster never experienced. The poor and poorest clusters were traditional, illustrated by the use of horses for transport. Men headed two-thirds of households, but the proportion headed by women was higher among the modern rich. Altogether, the results painted a picture of a traditional society in transition to becoming modern. The forerunners were educated, had more working members in the household, had fewer children, and were food secure but were not the richest according to the UBN characteristics, while those lagging were the poor, traditional, and food insecure.

The importance of food insecurity was illustrated by cluster 2, which ranked as fairly poor when using the UBN variables, becoming the most vulnerable in the multidimensional poverty analysis. The vulnerability was shown in cluster 2 by 62–64% of households reporting different levels of food insecurity, while almost 100% in cluster 5 never reported it.

It should be noted that the finding that participation in interventions, as getting water installed, receiving a microloan, or engaging in technical training coincided with better welfare as the modern clusters 5, 3, and 1 had most interventions.

The Health and Demographic Surveillance data have been judged to be of high quality (15, 16) and covered the whole population in the Cuatro Santos area with very few non-participants, thereby providing a reliable basis for analyses. The temporality of poverty predictors (a predictor happening before poverty) was not adequately captured by our design. Based on the dates of the initiation of the interventions stored in our database, we can, however, state that most interventions happened before the 2014 survey with most happening in 2005–2009 (16). The timing of acquisition of assets was not known, nor did we know exactly when the current head of the household was established, although analyses have shown stability over time regarding the household head. Food insecurity answers covered experiences during the last 4 weeks before the survey.

Cluster analysis is a powerful method to identify hidden groups in the data, and K-means is an algorithm that is fast and simple to use and interpret. Compared to some other clustering methods, the number of clusters can be visually selected on the scree plot. It is worth mentioning that the Euclidian distance was used, in which categorical variables were transformed into dummy variables, and the continuous variables were scaled. These metrics are very general and do not rely on any application assumptions. Our cluster analysis has, however, some limitations. Firstly, K-means clustering optimizes the distances to the cluster centroids, which means that spherical clusters are relatively easy to detect, but if a cluster has a complicated shape, K-means clustering might split this into two or more parts. Secondly, all variables were included in the distance measure of the cluster analysis, including potentially irrelevant variables. This might, in theory, lead to blurring of some clusters, although in our analysis, we managed to obtain well-interpretable clusters with clearly distinct properties. The interpretation and the choice of descriptive names of clusters were a subjective exercise that depended on the analyst's pre-understanding. The naming can, however, easily be reviewed by studying Supplemental Table 2, which displays the full cluster analysis.

Sufficient availability of food is among the basic capabilities, which was further emphasized by our findings showing food insecurity as essential for well-being in the multidimensional analysis of poverty. This was also reflected in the association between low self-rated health and food insecurity in a previous study from our group using data from the same surveillance system (24). Further, participation in interventions, such as water installation, microcredit, and participation in educational activities, was more common in clusters with better welfare, confirming our earlier results that these interventions were associated with poverty reduction (16).

Randomized controlled trials of multifaceted programs in six countries (25) and a recent evaluation with comparison areas for the Millennium Development Villages (26) reported effects on welfare by such complex interventions in poor areas. The Cuatro Santos case study (15) had no comparison areas so we cannot rule out that the secular trend in the Nicaraguan society explains the improvements in welfare seen in the area. The finding in this analysis of multiple dimensions of poverty, however, provides some support that the interventions contributed to poverty reduction.

The used Health and Demographic Surveillance data did not cover all aspects of basic capabilities. Even so, we captured multiple dimensions of poverty that are stressed by the capability approach. We consider the results to be meaningful, comprehensible, and familiar in the area, based on feedback and discussions held in the area with local community leaders and representatives of different sectors of society including health and security as well as laypeople from the communities. These local community representatives confirmed the usefulness of this and similar further analyses for targeting interventions intending to reduce inequity.

Applying cluster analysis to local data as in our case, the Cuatro Santos HDSS enables patterns of multidimensional poverty to be identified that are relevant for the studied context. Such identification can inform local policy aiming to amend identified characteristics among the households with the lowest well-being. In our case, the clusters identified having food insecurity and being less modern both inform on the type of interventions needed and identify characteristics of potential recipients. Such an analysis might be a powerful instrument for poverty reduction initiatives that would increase well-being.

Health and Demographic Surveillance data or similar rich data could, by using our outlined method, guide future studies in setting priority and direct interventions to increase general welfare. Another future task is developing computer applications where geographical information identifies the clustered households mapping them for direct interventions.

## Conclusion

The classification of households from rich to poor based on the UBN assessment was modified by a multidimensional analysis of poverty. The “fairly rich” households based on the UBN index were the forerunners of the modern lifestyle with higher welfare, while the fairly poor were the most food insecure. Results obtained from a cluster analysis may be useful for increased understanding of poverty. Health and Demographic Surveillance data and similar local data may be enhanced by computer applications for analysis and geographical mapping, which could guide local policy priority setting and direct interventions to increase general welfare.

## Data Availability Statement

The raw data supporting the conclusions of this article will be made available by the authors, without undue reservation, to any qualified researcher.

## Ethics Statement

The studies involving human participants were reviewed and approved by The Ethical Review Board of Biomedical Research at the National Autonomous University of León approved the HDSS data collection (FWA00004523/IRB0000334 ACTA No. 81). Written informed consent for participation was not required for this study in accordance with the national legislation and the institutional requirements.

## Author Contributions

CK managed the data, conducted the statistical analyses and drafted the manuscript, with support from KS. WP supported the data management. OS the statistical analyses. EB, MC, CK, RP, WP, and L-ÅP initiated and worked with the Cuatro Santos HDSS. All authors contributed and approved the final manuscript.

### Conflict of Interest

The authors declare that the research was conducted in the absence of any commercial or financial relationships that could be construed as a potential conflict of interest.
