# Supplementary material for: Assessing the Multiple Dimensions of Poverty. Data Mining Approaches to the 2004–14 Health and Demographic Surveillance System in Cuatro Santos, Nicaragua
Source: Front Public Health. 2020 Jan 29;7:409. doi: 10.3389/fpubh.2019.00409 (PMC7000462; doi:10.3389/fpubh.2019.00409)
Supplement: Supplementary file 1 [file Table_1.DOCX]

Supplemental Table 1. Cluster analysis output with the categories color marked as follows: Grey= Poverty assessed by the variables poverty and Unsatisfied Basic Needs (UBN), Light blue = variables in UBN, except head of household’s education, children’s school enrolment, and dependency ratio, Dark yellow = assets, Turquoise = food insecurity, Green = interventions, Light Yellow = derived individual variables.

|  | **Full Data** | **Cluster 0** | **Cluster 1** | **Cluster 2** | **Cluster 3** | **Cluster 4** | **Cluster 5** |
| --- | --- | --- | --- | --- | --- | --- | --- |
| N = 5253 (n)  % | (3466)  66 of N | (688)  20 | (540)  16 | (253)  7 | (752)  22 | (699)  20 | (534)  15 |
| Poor | 0.4616 | 0.9985 | 0 | 0.5178 | 0 | 0.9986 | 0.1573 |
| NonPoor | 0.5384 | 0.0015 | 1 | 0.4822 | 1 | 0.0014 | 0.8427 |
| ubn0 | 0.2216 | 0 | 0.3111 | 0.1502 | 0.3896 | 0 | 0.5037 |
| ubn1 | 0.3168 | 0.0015 | 0.6889 | 0.332 | 0.6104 | 0.0014 | 0.339 |
| ubn2 | 0.4103 | 0.8692 | 0 | 0.3953 | 0 | 0.9227 | 0.1479 |
| ubn3 | 0.0502 | 0.1265 | 0 | 0.1146 | 0 | 0.0758 | 0.0094 |
| ubn4 | 0.0012 | 0.0029 | 0 | 0.0079 | 0 | 0 | 0 |
| BrickWall | 0.2773 | 0.1221 | 0.3593 | 0.3399 | 0.4122 | 0.0873 | 0.4232 |
| AdobeWall | 0.7069 | 0.8416 | 0.6333 | 0.6245 | 0.5851 | 0.8941 | 0.573 |
| WoodWall | 0.0063 | 0.0174 | 0.0037 | 0.0119 | 0 | 0.0072 | 0 |
| PalmWall | 0.0006 | 0.0015 | 0 | 0.004 | 0 | 0 | 0 |
| CardbWall | 0.0078 | 0.016 | 0.0037 | 0.0158 | 0.0027 | 0.0114 | 0 |
| NoWall | 0.0012 | 0.0015 | 0 | 0.004 | 0 | 0 | 0.0037 |
| InPipe | 0.348 | 0.0291 | 0.6278 | 0.3992 | 0.5598 | 0.0229 | 0.5787 |
| ComPost | 0.0199 | 0.0102 | 0.0333 | 0.0079 | 0.0399 | 0.0014 | 0.0206 |
| OwnWell | 0.204 | 0.2791 | 0.1259 | 0.2095 | 0.137 | 0.3076 | 0.1423 |
| ComWell | 0.2969 | 0.4128 | 0.1519 | 0.2332 | 0.2114 | 0.4793 | 0.206 |
| River | 0.0791 | 0.1759 | 0.037 | 0.083 | 0.0239 | 0.1202 | 0.0187 |
| BuyWat | 0.0009 | 0 | 0 | 0.004 | 0 | 0.0014 | 0.0019 |
| WaterOth | 0.0514 | 0.093 | 0.0241 | 0.0632 | 0.0279 | 0.0672 | 0.0318 |
| CeramFloor | 0.0802 | 0 | 0.063 | 0.004 | 0.1676 | 0.0057 | 0.2116 |
| BrickFloor | 0.0542 | 0.0015 | 0.0778 | 0.0119 | 0.0838 | 0.0014 | 0.1461 |
| MudBrickFloor | 0.0069 | 0 | 0.0148 | 0.0079 | 0.008 | 0 | 0.015 |
| TilingFloor | 0.2931 | 0.0218 | 0.5278 | 0.2846 | 0.5505 | 0.0272 | 0.3951 |
| MudFloor | 0.5655 | 0.9767 | 0.3167 | 0.6917 | 0.1902 | 0.9657 | 0.2322 |
| Toilet | 0.0239 | 0 | 0.0185 | 0.0158 | 0.0465 | 0 | 0.0637 |
| Latrine | 0.7822 | 0.6759 | 0.8722 | 0.7589 | 0.8617 | 0.7024 | 0.8315 |
| NoLatrine | 0.1939 | 0.3241 | 0.1093 | 0.2253 | 0.0918 | 0.2976 | 0.1049 |
| Light | 0.8979 | 0.7703 | 0.9611 | 0.8458 | 0.9814 | 0.8412 | 0.9794 |
| NoLight | 0.1021 | 0.2297 | 0.0389 | 0.1542 | 0.0186 | 0.1588 | 0.0206 |
| Gasstove | 0.088 | 0.0058 | 0.0463 | 0 | 0.1503 | 0.0129 | 0.2884 |
| ImprovedStove | 0.0144 | 0.016 | 0.0148 | 0 | 0.0253 | 0.0072 | 0.0131 |
| NormalStove | 0.8883 | 0.968 | 0.9296 | 0.9763 | 0.8178 | 0.9728 | 0.691 |
| NoStove | 0.0092 | 0.0102 | 0.0093 | 0.0237 | 0.0066 | 0.0072 | 0.0075 |
| Parabol | 0.1145 | 0.0073 | 0.1 | 0.0237 | 0.1848 | 0.03 | 0.3221 |
| NormalAntenna | 0.3924 | 0.2442 | 0.437 | 0.2925 | 0.5053 | 0.382 | 0.4401 |
| HandmadeAntenna | 0.0837 | 0.1235 | 0.1056 | 0.1225 | 0.0731 | 0.0687 | 0.0262 |
| NoAntenna | 0.4094 | 0.625 | 0.3574 | 0.5613 | 0.2367 | 0.5193 | 0.2116 |
| CarYes | 0.026 | 0 | 0.0259 | 0 | 0.0332 | 0.0014 | 0.0936 |
| CarNo | 0.974 | 1 | 0.9741 | 1 | 0.9668 | 0.9986 | 0.9064 |
| MCYes | 0.0822 | 0.0087 | 0.0519 | 0.0158 | 0.1423 | 0.0243 | 0.2303 |
| MCNo | 0.9178 | 0.9913 | 0.9481 | 0.9842 | 0.8577 | 0.9757 | 0.7697 |
| BikeYes | 0.1558 | 0.0698 | 0.1426 | 0.1146 | 0.2261 | 0.1373 | 0.2247 |
| BikeNo | 0.8442 | 0.9302 | 0.8574 | 0.8854 | 0.7739 | 0.8627 | 0.7753 |
| HorseYes | 0.2574 | 0.327 | 0.1981 | 0.249 | 0.2287 | 0.2804 | 0.2416 |
| HorseNo | 0.7426 | 0.673 | 0.8019 | 0.751 | 0.7713 | 0.7196 | 0.7584 |
| Fridge | 0.2995 | 0.0959 | 0.2852 | 0.1304 | 0.492 | 0.1173 | 0.6236 |
| NoFridge | 0.7005 | 0.9041 | 0.7148 | 0.8696 | 0.508 | 0.8827 | 0.3764 |
| SewingMach | 0.0623 | 0.0465 | 0.0463 | 0.0474 | 0.0931 | 0.0315 | 0.103 |
| NoSewingMach | 0.9377 | 0.9535 | 0.9537 | 0.9526 | 0.9069 | 0.9685 | 0.897 |
| Computer | 0.0343 | 0 | 0.0259 | 0.0079 | 0.0479 | 0.0043 | 0.1199 |
| NoComputer | 0.9657 | 1 | 0.9741 | 0.9921 | 0.9521 | 0.9957 | 0.8801 |
| BreadOven | 0.1725 | 0.1802 | 0.1759 | 0.2016 | 0.1636 | 0.1803 | 0.1479 |
| NoBreadOven | 0.8275 | 0.8198 | 0.8241 | 0.7984 | 0.8364 | 0.8197 | 0.8521 |
| Chimney | 0.0193 | 0.0218 | 0.0222 | 0.0198 | 0.0266 | 0.0143 | 0.0094 |
| NoChimney | 0.9807 | 0.9782 | 0.9778 | 0.9802 | 0.9734 | 0.9857 | 0.9906 |
| FI1Never | 0.1405 | 0.0276 | 0.0167 | 0 | 0.0426 | 0.0601 | 0.721 |
| FI1Rarely | 0.3912 | 0.0872 | 0.0944 | 0.0079 | 0.8125 | 0.7625 | 0.1854 |
| FI1Sometimes | 0.2475 | 0.5058 | 0.5926 | 0.0316 | 0.0957 | 0.1116 | 0.0599 |
| FI1Often | 0.2207 | 0.3794 | 0.2963 | 0.9605 | 0.0492 | 0.0658 | 0.0337 |
| FI2Never | 0.1362 | 0.0102 | 0.0056 | 0.004 | 0.0213 | 0.0386 | 0.7828 |
| FI2Rarely | 0.4192 | 0.0218 | 0.0241 | 0.0119 | 0.9269 | 0.9027 | 0.176 |
| FI2Sometimes | 0.3439 | 0.8939 | 0.9185 | 0.0356 | 0.0346 | 0.0386 | 0.0356 |
| FI2Often | 0.1007 | 0.0741 | 0.0519 | 0.9486 | 0.0173 | 0.02 | 0.0056 |
| FI3Never | 0.1919 | 0.0552 | 0.0389 | 0.004 | 0.0625 | 0.0916 | 0.9251 |
| FI3Rarely | 0.4553 | 0.2238 | 0.2537 | 0.0119 | 0.887 | 0.8412 | 0.0543 |
| FI3Sometimes | 0.2767 | 0.7064 | 0.6944 | 0.0514 | 0.0412 | 0.0644 | 0.0169 |
| FI3Often | 0.0762 | 0.0145 | 0.013 | 0.9328 | 0.0093 | 0.0029 | 0.0037 |
| FI4Never | 0.176 | 0.0363 | 0.0315 | 0.0198 | 0.0279 | 0.0658 | 0.9288 |
| FI4Rarely | 0.4896 | 0.3052 | 0.3019 | 0.0198 | 0.9255 | 0.8541 | 0.0487 |
| FI4Sometimes | 0.2701 | 0.6439 | 0.637 | 0.1937 | 0.0452 | 0.0773 | 0.0225 |
| FI4Often | 0.0643 | 0.0145 | 0.0296 | 0.7668 | 0.0013 | 0.0029 | 0 |
| FI5Never | 0.2545 | 0.1061 | 0.1056 | 0.0158 | 0.1529 | 0.1588 | 0.9775 |
| FI5Rarely | 0.474 | 0.3968 | 0.3556 | 0.0514 | 0.8125 | 0.7825 | 0.0131 |
| FI5Sometimes | 0.2227 | 0.4927 | 0.5296 | 0.3123 | 0.0346 | 0.0544 | 0.0075 |
| FI5Often | 0.0488 | 0.0044 | 0.0093 | 0.6206 | 0 | 0.0043 | 0.0019 |
| FI6Never | 0.3883 | 0.2311 | 0.2796 | 0.0474 | 0.3777 | 0.3019 | 0.9906 |
| FI6Rarely | 0.3996 | 0.3808 | 0.3519 | 0.1344 | 0.5904 | 0.6466 | 0.0056 |
| FI6Sometimes | 0.1769 | 0.3852 | 0.3593 | 0.3794 | 0.0306 | 0.0472 | 0.0037 |
| FI6Often | 0.0352 | 0.0029 | 0.0093 | 0.4387 | 0.0013 | 0.0043 | 0 |
| FI7Never | 0.7074 | 0.6308 | 0.6759 | 0.253 | 0.7793 | 0.6724 | 0.9981 |
| FI7Rarely | 0.2152 | 0.2282 | 0.2241 | 0.3597 | 0.2141 | 0.3076 | 0.0019 |
| FI7Sometimes | 0.0666 | 0.1381 | 0.1 | 0.253 | 0.0066 | 0.0186 | 0 |
| FI7Often | 0.0107 | 0.0029 | 0 | 0.1344 | 0 | 0.0014 | 0 |
| FI8Never | 0.8474 | 0.7515 | 0.8185 | 0.336 | 0.9481 | 0.9242 | 1 |
| FI8Rarely | 0.1119 | 0.1788 | 0.1481 | 0.3953 | 0.0505 | 0.0672 | 0 |
| FI8Sometimes | 0.0366 | 0.0683 | 0.0315 | 0.2213 | 0.0013 | 0.0086 | 0 |
| FI8Often | 0.004 | 0.0015 | 0.0019 | 0.0474 | 0 | 0 | 0 |
| FI9Never | 0.899 | 0.8445 | 0.863 | 0.5257 | 0.9721 | 0.9599 | 1 |
| FI9Rarely | 0.073 | 0.1105 | 0.113 | 0.2925 | 0.0266 | 0.0315 | 0 |
| FI9Sometimes | 0.0248 | 0.0451 | 0.0241 | 0.1383 | 0.0013 | 0.008 | 0 |
| FI9Often | 0.0032 | 0 | 0 | 0.0435 | 0 | 0 | 0 |
| WaterMeter | 0.2132 | 0.0116 | 0.3574 | 0.2372 | 0.3763 | 0.0172 | 0.3427 |
| NoWaterMeter | 0.7868 | 0.9884 | 0.6426 | 0.7628 | 0.6237 | 0.9828 | 0.6573 |
| Microloan | 0.1269 | 0.0392 | 0.1593 | 0.0672 | 0.2021 | 0.0701 | 0.2041 |
| NoMicroloan | 0.8731 | 0.9608 | 0.8407 | 0.9328 | 0.7979 | 0.9299 | 0.7959 |
| Garden | 0.0641 | 0.0291 | 0.1037 | 0.1028 | 0.0691 | 0.0658 | 0.0412 |
| NoGarden | 0.9359 | 0.9709 | 0.8963 | 0.8972 | 0.9309 | 0.9342 | 0.9588 |
| UseGarden | 0.0378 | 0.0116 | 0.0556 | 0.0553 | 0.0412 | 0.0486 | 0.0262 |
| NoUseGarden | 0.9622 | 0.9884 | 0.9444 | 0.9447 | 0.9588 | 0.9514 | 0.9738 |
| Training | 0.0961 | 0.0538 | 0.0852 | 0.0593 | 0.125 | 0.073 | 0.1685 |
| NoTraining | 0.9039 | 0.9462 | 0.9148 | 0.9407 | 0.875 | 0.927 | 0.8315 |
| HHchildren | 1.7074 | 1.7892 | 1.7426 | 1.9091 | 1.6702 | 1.7797 | 1.4288 |
| HHadults | 4.7496 | 4.6337 | 5.0148 | 5.17 | 4.8989 | 4.5136 | 4.53 |
| HHnotworking | 2.6218 | 2.6308 | 2.8778 | 2.8379 | 2.6396 | 2.4936 | 2.3914 |
| HHworking | 1.4106 | 1.3968 | 1.4111 | 1.4585 | 1.4069 | 1.4206 | 1.397 |
| HHadultsworking | 1.3947 | 1.3779 | 1.3963 | 1.4387 | 1.3989 | 1.3934 | 1.3895 |
| HHadultsnotworking | 1.6691 | 1.5945 | 1.8648 | 1.7826 | 1.7487 | 1.5265 | 1.588 |
| HHindividuals | 6.457 | 6.423 | 6.7574 | 7.0791 | 6.5691 | 6.2933 | 5.9588 |
| HHRW | 1.6083 | 1.3876 | 1.4179 | 1.4572 | 1.6796 | 1.66 | 1.9887 |
| HHDep | 0.247 | 0.2403 | 0.2273 | 0.2235 | 0.249 | 0.2566 | 0.2713 |
| NoDeaths | 0.9423 | 0.9448 | 0.9481 | 0.9249 | 0.9468 | 0.9299 | 0.9513 |
| Deaths | 0.0577 | 0.0552 | 0.0519 | 0.0751 | 0.0532 | 0.0701 | 0.0487 |
| NoBirths | 0.7403 | 0.7471 | 0.7167 | 0.7036 | 0.7434 | 0.7511 | 0.7547 |
| Births | 0.2597 | 0.2529 | 0.2833 | 0.2964 | 0.2566 | 0.2489 | 0.2453 |
| NoImmigr | 0.6036 | 0.6512 | 0.6167 | 0.6206 | 0.5864 | 0.5465 | 0.6199 |
| Immigr | 0.3964 | 0.3488 | 0.3833 | 0.3794 | 0.4136 | 0.4535 | 0.3801 |
| NoEmigr | 0.4365 | 0.4564 | 0.3907 | 0.3913 | 0.3963 | 0.5093 | 0.4401 |
| Emigr | 0.5635 | 0.5436 | 0.6093 | 0.6087 | 0.6037 | 0.4907 | 0.5599 |
| FemHead | 0.2634 | 0.2253 | 0.3 | 0.2767 | 0.2806 | 0.2361 | 0.2809 |
| MaleHead | 0.7366 | 0.7747 | 0.7 | 0.7233 | 0.7194 | 0.7639 | 0.7191 |
| NoIllit | 0.7248 | 0.6061 | 0.7426 | 0.5692 | 0.7965 | 0.6924 | 0.8745 |
| Illit | 0.2752 | 0.3939 | 0.2574 | 0.4308 | 0.2035 | 0.3076 | 0.1255 |
| NoEduc | 0.0395 | 0.0741 | 0.0296 | 0.0632 | 0.0146 | 0.0558 | 0.0075 |
| PrimEduc | 0.3177 | 0.4927 | 0.2352 | 0.4585 | 0.2154 | 0.4192 | 0.1199 |
| SecEduc | 0.4394 | 0.3605 | 0.5093 | 0.3597 | 0.4894 | 0.4464 | 0.4288 |
| TechEduc | 0.073 | 0.0349 | 0.0889 | 0.0435 | 0.0957 | 0.0343 | 0.1386 |
| UnivEduc | 0.1304 | 0.0378 | 0.137 | 0.0751 | 0.1848 | 0.0443 | 0.3052 |
| WomHGood | 0.5698 | 0.5538 | 0.5315 | 0.5099 | 0.5678 | 0.5937 | 0.6292 |
| WomHBad | 0.4302 | 0.4462 | 0.4685 | 0.4901 | 0.4322 | 0.4063 | 0.3708 |
| NoForeignIm | 0.9371 | 0.9564 | 0.9352 | 0.9486 | 0.9149 | 0.9428 | 0.9326 |
| ForeignIm | 0.0629 | 0.0436 | 0.0648 | 0.0514 | 0.0851 | 0.0572 | 0.0674 |
| NoForeignEm | 0.8661 | 0.8924 | 0.8759 | 0.8775 | 0.8351 | 0.8913 | 0.8277 |
| ForeignEm | 0.1339 | 0.1076 | 0.1241 | 0.1225 | 0.1649 | 0.1087 | 0.1723 |
| NoChildWork | 0.9844 | 0.9811 | 0.9852 | 0.9802 | 0.9934 | 0.9728 | 0.9925 |
| ChildWork | 0.0156 | 0.0189 | 0.0148 | 0.0198 | 0.0066 | 0.0272 | 0.0075 |
| NoHomeBirth | 0.9804 | 0.9724 | 0.9833 | 0.9921 | 0.984 | 0.9728 | 0.9869 |
| HomeBirth | 0.0196 | 0.0276 | 0.0167 | 0.0079 | 0.016 | 0.0272 | 0.0131 |
| NoHospitalBirth | 0.7882 | 0.8169 | 0.7741 | 0.8379 | 0.7819 | 0.7811 | 0.7603 |
| NoCHCBirth | 0.9348 | 0.9172 | 0.9426 | 0.917 | 0.9481 | 0.9156 | 0.9644 |
| CHCBirth | 0.0652 | 0.0828 | 0.0574 | 0.083 | 0.0519 | 0.0844 | 0.0356 |
| HospitalBirth | 0.2118 | 0.1831 | 0.2259 | 0.1621 | 0.2181 | 0.2189 | 0.2397 |
| NoU5D | 0.9902 | 0.9869 | 0.9926 | 0.9881 | 0.9947 | 0.9857 | 0.9925 |
| U5D | 0.0098 | 0.0131 | 0.0074 | 0.0119 | 0.0053 | 0.0143 | 0.0075 |
