# Supplementary material for: Assessing the Multiple Dimensions of Poverty. Data Mining Approaches to the 2004–14 Health and Demographic Surveillance System in Cuatro Santos, Nicaragua
Source: Front Public Health. 2020 Jan 29;7:409. doi: 10.3389/fpubh.2019.00409 (PMC7000462; doi:10.3389/fpubh.2019.00409)

Supplemental figure 1. Scree plot displaying within cluster Sums of Squared Errors (y-axis) and number of clusters (x-axis) from K-means cluster analysis of data from Cuatro Santos Health and Demographic Surveillance System, 2014

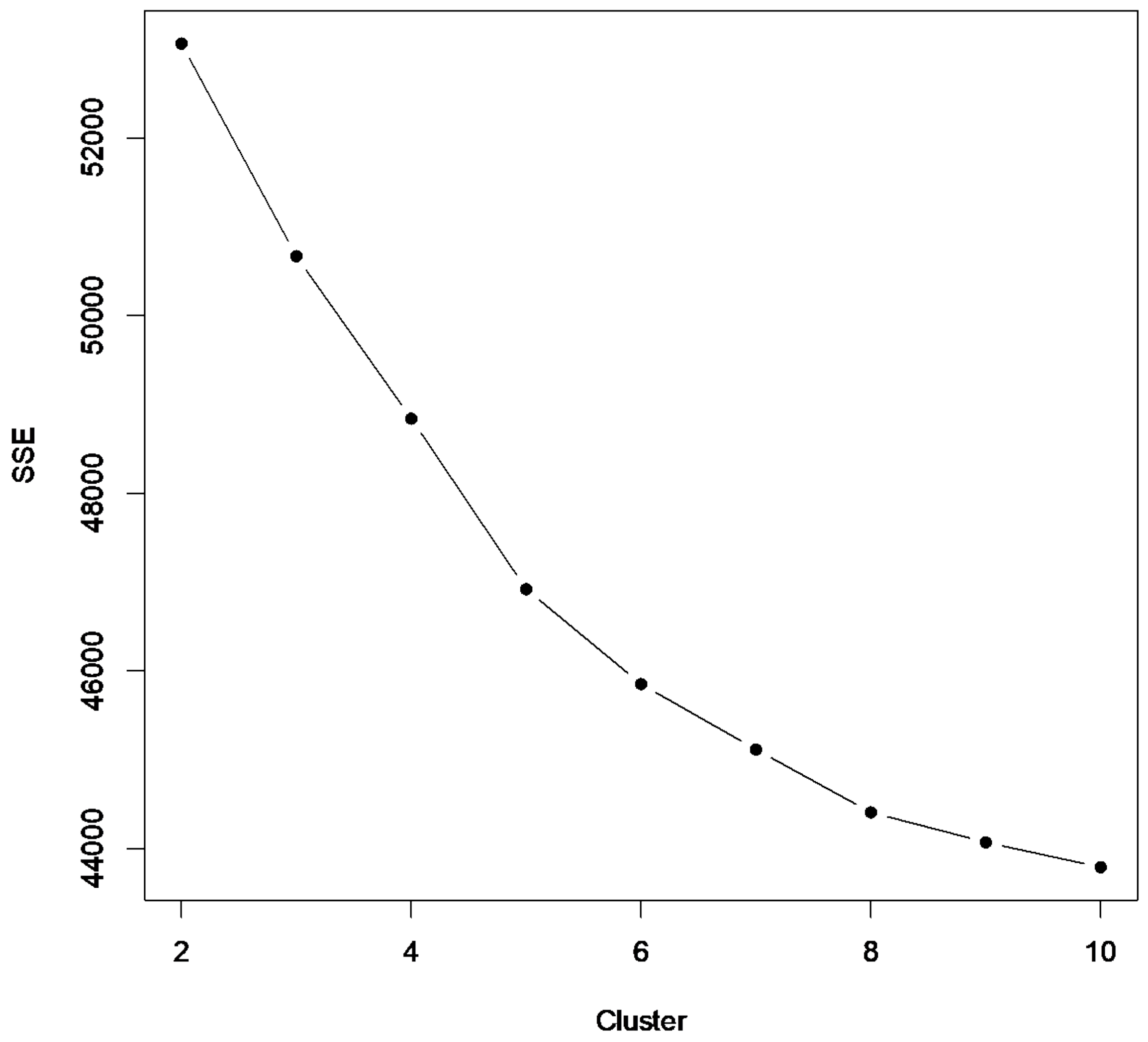

Supplement: Supplementary file 2 [file Image_1.pdf]
